# Cholagogues

**Published:** 1879-10

**Authors:** 


CHOLAGOGUES.

A COMMISSION, with Dr. Rutherford, of Edinburgh, at its head, has instituted an investigation into the action of certain cholagogues upon the secretion of bile. Its conclusions concerning the action of these important remedies, in daily use by the profession, are of great interest. The experiments were made upon dogs. The effects, thus obtained, may not be exactly similar to the action of these agents in the human system.

Podophyllin, aloes, rheum, euonymin, sanguinarin, iridin, leptandrin, colocynth, jalap, sodium phosphate, mercuric chloride, phytolaccin, hydrastin, juglandin, sodium and ammonium benzoate, benzoic acid, sodium salicylate, and nitro-muriatic acid, all are more or less powerful hepatic stimulants, positively increasing the amount of bile secreted, and all, except the last five, being more or less stimulant, and irritating to the intestinal glands. Senna, colchicum, taraxacum and Scammony are feeble hepatic stimulants. Gamboge, castor oil and calomel stimulate the intestinal glands, but not the liver. Ipecacuanha is simply a hepatic stimulant. Magnesium sulphate is only stimulant to the intestines, as are also ammonium chloride and menispermin.

Calabar bean stimulates the liver, and atropia antagonizes this action, but atropia administered alone does not affect the secretion of bile.

It is also found in man that four grains of iridin at night is a certain remedy for "biliousness." Euonymin, in two grain doses, also has the same effect. Both are liable to be followed by depression, if the dose be too large, and both should be followed by a saline aperient in the morning.—*British Medical Journal, Feb. 8, 1879.*

